# Osteoimmunology and Bone Homeostasis: Relevance to Spondyloarthritis

**DOI:** 10.1007/s11926-013-0342-2

**Published:** 2013-05-25

**Authors:** Steven R. Goldring

**Affiliations:** The Hospital for Special Surgery, Weill Cornell Medical College, 535 East 70th Street, New York, NY 10128 USA

**Keywords:** Osteoimmunology, Bone homeostasis, Spondyloarthritis, Ankylosing spondylitis, Psoriatic arthritis, Osteoclast, Osteoblast, Osteocyte, Bone remodeling, Inflammation, Cytokine, Bone morphogenic proteins, Wnt/βcatenin, Sclerostin

## Abstract

The seronegative spondyloarthopathies (SpA) share certain common articular and peri-articular features that differ from rheumatoid arthritis (RA) and other forms of inflammatory arthritis. These include the tendency of the SpAs to involve the axial skeleton in addition to the diarthrodial joints, and the prominent involvement of the extra-articular entheses (sites of ligamentous and tendon insertion), which are not common sites of primary pathology in RA and other inflammatory arthropathies. The differential anatomic sites of bone pathology in the SpAs in comparison to the other forms of arthritis suggest that the underlying pathogenic processes and cellular and molecular mechanisms that account for the peri-articular bone pathology involve different underlying disease mechanisms. This review will highlight the molecular and cellular processes that are involved in the pathogenesis of the skeletal pathology in the SpAs, and provide evidence that many of the factors involved in regulation of bone cell function exhibit potent immune-regulatory activity, providing support for the general concept of osteoimmunology.

## Introduction

The seronegative spondyloarthopathies (SpA) that include ankylosing spondylitis (AS), reactive arthritis, and arthritis associated with psoriasis or inflammatory bowel disease, and juvenile onset spondyloarthropathy, share certain common articular and peri-articular features that differ from rheumatoid arthritis (RA) and other forms of inflammatory arthritis. These differences include the tendency of the SpAs to involve the axial skeleton in addition to the diarthrodial joints [1, 2], and the prominent involvement of the extra-articular entheses (sites of ligamentous and tendon insertion), which are not common sites of primary pathology in RA and other inflammatory arthropathies. The differential anatomic sites of the articular and peri-articular bone pathology in the SpAs in comparison to the other forms of arthritis suggests that the underlying pathogenic processes that account for the peri-articular inflammation involve different underlying disease mechanisms. Analysis of the inflamed tissues at sites of skeletal pathology support this hypothesis, in that the cell types and profiles of inflammatory mediators differ in the SpAs in comparison to RA and other forms of inflammatory arthritis. Many of the mediators involved in the de-regulation of bone cell activity in the SpAs and RA also function as potent regulators of the immune system. This observation has led to the development of a specialized field of investigation that is generally identified as “osteoimmunology”. This review will highlight the molecular and cellular processes that are involved in the pathogenesis of the skeletal pathology in the SpAs, and provide evidence that many of the factors involved in regulation of bone cell function exhibit potent immune-regulatory activity, providing support for the general concept of osteoimmunology.

## Mechanisms of Physiological Bone Adaptation and Remodeling

Peri-articular bone is organized into distinct structural entities that include the subchondral bone plate, which is comprised of compact cortical bone, and a deeper zone of contiguous trabecular bone that is encased within the bone marrow. The bone that forms the joint margins and extends distally from the joint surface is also comprised of compact bone that is lined by the periosteum. The peri-articular bone at all of these sites undergoes marked changes in response to biological and biomechanical signals produced by the local inflammatory processes associated with the SpAs. These bone changes are mediated by bone cells that modify the architecture and properties of the bone through active cellular processes of modeling and remodeling [3]. Modeling is a process that involves direct osteoblast-mediated apposition of bone to existing bone surfaces without a resorptive phase. Remodeling involves the recruitment of monocytic osteoclast precursors to the bone surface followed by differentiation of these cells into osteoclasts that are uniquely adapted to removal of the mineralized bone matrix. Multiple lines of evidence have established that the osteoclast is required for resorption of bone under physiologic conditions of bone remodeling [4, 5]. The role of the osteoclast in pathologic states associated with SpA and other forms of inflammatory arthritis will be discussed in a subsequent section.

In the remodeling cycle, the phase of osteoclast-mediated bone resorption is followed by a so-called reversal phase after which the resorbed bone surface is populated by precursors of the osteoblast, which are of mesenchymal origin. The osteoblast lineage cells then undergo differentiation into mature bone-forming osteoblasts that replace the resorbed bone. Remodeling occurs during embryonic development and throughout post-natal life, and provides a cellular system for adapting bone to biomechanical influences, repairing damage to the bone matrix and, under certain conditions, releasing calcium for maintenance of mineral ion homeostasis. Importantly, under physiological conditions, the amount of bone that is removed during the phase of bone resorption is exactly matched by the amount of bone that is added during the phase of bone formation, such that bone mass is preserved during the remodeling cycle. The mechanisms that are involved in the “coupling” of the bone resorption and formation are not fully understood. Both transforming factor-β (TGF-β) and bone morphogenic proteins (BMP) are growth factors that are sequestered within the bone matrix during osteoblast-mediated bone formation, and their release during the phase of osteoclast-mediated bone resorption represents a potential mechanism for linking the resorptive process to bone formation [6, 7]. There is also evidence that osteoclasts may themselves be the source of factors that can function either as inhibitors or activators of osteoblast differentiation and activity [8•, 9]. In the SpAs, the so-called “coupling” of bone formation and resorption is de-regulated such that there is localized loss of bone in the entheseal insertion sites and excessive bone formation in periosteal sites adjacent to the sites of bone erosion.

Etheseal inflammation is a unique feature of the SpAs that results in characteristic skeletal pathological changes. At these sites, the new bone is added by a process of endochondral ossification that recapitulates the cellular mechanisms of bone growth that occur during skeletal growth and development in which new bone is formed by replacement of a cartilaginous matrix. Syndesmophytes, which are one of the radiographic hallmarks of the SpAs, represent examples of ossification at the margins of vertebral bodies that are formed via the process of endochondral ossification [10]. Local production of bone growth factors, including TGF-β and BMPs, which play a role in endochonral bone formation during development and post-natally in fracture repair, have been implicated in this process [10–14].

Recent studies have identified the role of the osteocyte in contributing to the regulation of bone remodeling [15•, 16•]. These cells are the most abundant bone cell type. They are embedded within the bone matrix where they form an interconnected network with each other and with the cells on the bone surface. In response to mechanical signals, local soluble mediators and systemic hormones release products that control the activities of the osteoclasts and osteoblasts involved in the remodeling process. Two of these osteocyte-derived products, receptor activator of NF-κB ligand (RANKL) and sclerostin, play essential roles in regulating bone remodeling. RANKL was originally identified as TNF-related activation-induced cytokine (TRANCE) that regulated dendritic cell activity and played a role in development of the immune system [17, 18]. Subsequently, RANKL was shown to be a master regulator of osteoclast differentiation that was required for formation of osteoclasts and bone resorption [19, 20]. Production of RANKL by osteocytes enhances osteoclast-mediated bone resorption in response to mechanical stimuli and hormones such as parathyroid hormone [15•, 16•].

Sclerostin is a product of the *sost* gene, which is a potent inhibitor of the Wnt/βcatenin signaling pathway that contributes to the regulation of bone formation [21]. The expression of this gene is regulated in part by mechanical factors and soluble mediators such as prostaglandins [22•, 23, 24]. The Dickkopf (DKK) family of proteins, which are produced by a variety of cell types, also inhibit bone formation by interfering with signaling by the Wnt/βcatenin pathway [21, 25•]. Both DKK-1 and sclerostin, as well as soluble frizzled related protein (sFRP), which binds Wnt ligands involved in activation of the Wnt/βcatenin pathway, are implicated in the suppression of bone repair in RA. As will be discussed in the following section, the reduced production of these inhibitors of bone formation likely contributes to the excessive bone formation that characterizes the skeletal pathology in the SpAs.

## Mechanisms of Pathological Bone Remodeling in the SpAs

Investigators have utilized MRI and anatomic and histopathological analysis to examine the pathological changes at the entheses, which are primary sites of inflammation in the SpAs [12, 26, 27, 28•]. The inflammatory tissue often extends to the synovial lining, which exhibits synovial hyperplasia, lymphocytic infiltration, and pannus formation. The development of synovial pannus and entheseal inflammation is also accompanied by inflammatory cell infiltration in the subchondral bone marrow [29]. Cells with morphological features of osteoclasts can be identified at the interface between the bone surface and the inflammatory tissue associated with bone erosions, providing evidence that, similar to RA, osteoclasts are the cell type responsible for the pathological bone resorption. Studies employing animal models of inflammatory arthritis in which osteoclast formation is defective have helped to provide strong evidence that osteoclasts are the principal cell type responsible for the pathological bone resorption in inflammatory arthropathies. Pettit et al. generated inflammatory arthritis in mice lacking the gene for RANKL [30]. They showed that the RANKL knock-out mice, which lacked the capacity to form osteoclasts, were protected from the development of bone erosions despite a level of synovial inflammation comparable to the wild-type mice. Subsequently, other investigators [31, 32] provided further evidence demonstrating the requirement for osteoclasts in the pathogenesis of bone erosions utilizing additional murine models of inflammatory arthritis.

As described in the preceding discussion, a striking feature of the skeletal pathology in the SpAs is the presence of enhanced bone formation associated with the entheseal and synovial pathology. Braun [12] analyzed tissues from inflamed sacroiliac joints obtained from patients with AS, and noted the presence of dense infiltrates of CD14 positive macrophages and CD4^+^ and CD8T^+^ lymphocytes. They also observed localized nodules of endochondral ossification within the inflamed tissues. Analysis, using in situ hybridization, revealed that many of the inflammatory cells expressed abundant messenger RNA for tumor necrosis factor-α (TNF-α), but not interleukin IL-1β (IL-1β). Of interest, TGF-β_2_ message was noted in close proximity to the regions of new bone formation. More recently, Lories et al. [13] examined synovial biopsies from patients with AS and RA and detected high levels of BMP-2 and -6 expression in synovial fibroblasts and macrophages. They also showed that treatment of synovial fibroblasts with TNF-α or IL-1 increased BMP-2 and BMP-6 expression, providing a potential mechanism for the enhanced production of these bone growth factors in the inflamed synovial tissues. The detection of the bone growth factors in the RA synovium was surprising in light of the absence of new bone formation at sites of synovial pathology in RA, and they speculated that the suppression of bone repair could be related to the production in the RA synovium of potent factors that inhibit osteoblast-mediated bone formation.

Diarra and co-workers [33] have provided insights into the mechanism responsible for the suppressed bone formation and repair associated with the synovial lesion in RA using animal models of RA. Analysis of inflamed synovial tissues in animals with arthritis revealed high levels of expression of DKK-1, the potent inhibitor of the Wnt/βcatenin pathway. They also analyzed synovial tissue from RA patients and found that DKK-1 was expressed in synovial fibroblasts, endothelial cells, and in chondrocytes located in cartilage immediately adjacent to sites of pannus invasion. Using in vitro cell cultures they showed that TNF-α markedly increased the production of DKK-1 in cultured synovial fibroblasts. Analysis of the serum levels of DKK-1 in RA patients revealed that DKK-1 levels were elevated in RA patients compared to healthy controls, and that DKK-1 levels correlated with measures of clinical disease activity. In contrast, in patients with AS, the serum DKK-1 levels were below the levels in the healthy control population. In studies by Diarra et al. [33], treatment of animals with arthritis with an antibody to DKK-1 resulted in increased bone repair, but in addition they unexpectedly observed a marked inhibition in focal articular bone resorption. The effect on bone resorption was attributed to an increase in the production of osteoprotegerin (OPG), the potent inhibitor of RANKL. They speculated that the increase in OPG was related to inhibition of DKK-1 and resultant up-regulation of the Wnt pathway that led to activation of βcatenin, which transcriptionally enhances OPG gene expression [33, 34].

Walsh et al. extended the observations of Diarra using the serum transfer model of arthritis [35]. Quantitative polymerase chain reaction was used to evaluate the expression levels of a spectrum of Wnt pathway inhibitors in retrieved synovial specimens from arthritic and wild type animals. They showed that the levels of several members of the DKK-1 family, as well as the soluble frizzled related protein (sFRP), were elevated in the synovial tissues from animals with arthritis compared to the controls, and employing immunostaining confirmed the local expression of DKK-1 and sFRP1 in synovial tissue at sites of erosions.

Similar to the observations of Diarra et al. [33], Appel and co-workers [11] found that serum levels of DKK-1 were reduced in patients with AS compared to healthy controls. In addition, using serial samples, they showed serum sclerostin levels correlated with the propensity to form syndesmophytes and noted that low serum sclerostin levels were associated with the formation of new syndesmophytes. They also analyzed bone tissue from joints from patients with AS and noted the virtual absence of sclerostin expression in osteocytes at sites of new bone formation, providing further evidence that the lack of suppression of the Wnt/βcatenin signaling contributed to the enhanced bone formation.

A recent publication by Sherlock et al. [28•] provides further evidence of the unique interplay between the bone and immune systems. Employing a murine model of AS, the authors identified the presence of T cells that were responsive to IL-23 in the entheses. IL-23 is a cytokine that previously had been linked to the pathogenesis of both articular and extra-articular inflammation in the SpAs [36, 37]. Importantly, they showed that overexpression of IL-23 was sufficient to induce a destructive enthesitis with histopathologic and bone remodeling features that pheno-copied the features of AS. They found that both IL-17 and IL-22 contributed to the pathology, and that IL-22 was the predominant effector molecule. They further showed that IL-22 could induce osteoblast differentiation via effects on the Wnt and BMP pathways, providing a link between the proinflammatory and immunomodulatory activities of this cytokine and its capacity to modulate bone remodeling and enhance bone formation at sites of inflammation. The bone anabolic effects of IL-22 are not unique to this cytokine, and other proinflammatory cytokines also have been shown to have pro-osteogenic effects. These include, IL-1, IL-17, IL-18, IL-33 and oncostatin M, which under certain conditions can enhance osteoblast differentiation [38–41]. Although the specific mediators have not been rigorously defined, T cells and monocyte–macrophage lineage cells have also been shown to exert pro-osteogenic effects on osteoblast precursors, providing additional evidence that under certain conditions immune cells and their products may contribute not only to catabolic processes but also participate in anabolic tissue responses, at least with respect to bone [42–45].

MRI imaging has been extensively utilized to study the inflammatory bone pathology associated with the SpAs. In these studies, so-called osteitis is manifest by the presence of an increased signal on STIR sequences. The altered signals have been identified in multiple skeletal sites, including the sacroiliac and zygapophyseal joints and the vertebral bodies, where they are most often localized to the endplates or corners of the vertebral bodies [46]. Histopathological examination of tissues from these sites has confirmed the presence of inflammatory cell infiltration with monocyte macrophages and T and B cells accompanied by variable degrees of edema [47–49].

Multiple previously published studies in patients with AS have observed that, although anti-TNF-α therapy effectively suppresses entheseal and synovial inflammation, the treatment does not prevent progression of syndesmophyte formation [50–53]. Some authors have interpreted these findings to suggest that the formation of syndesmophytes following resolution of inflammation after anti-TNF therapy supports the theory that TNF-α acts as a brake on bone formation, and that blockade of its activity releases this constraint and allows new bone formation [54–56]. An alternate explanation is that inflammation and new bone formation may be independent of each other, and that the pathogenic processes responsible for the enhanced bone formation may escape control by the therapies such as anti-TNF-α that suppresses the inflammatory process.

A recent publication by Baraliakos et al. [57•] has provided data on the long-term follow-up of patients with AS treated successfully with anti-TNF therapy. They compared the radiographic progression of syndesmophytes in AS patients treated with infliximab versus historical controls who had not received TNF-blockers over 8 years. Although there were limitations in the study design, their data indicated that there was less new bone formation in patients treated with anti-TNF therapy, and they suggested that the processes of inflammation and new bone formation were in fact linked. They speculated that this argued against the hypothesis of a mechanism for the development of excessive new bone formation that was independent of inflammation.

Paradoxically, although individuals with SpA exhibit localized regions of enhanced bone formation at sites of spinal inflammation, many patients have evidence of marked osteopenia of the spinal column. Studies indicate that disease burden correlates with the osteoporosis and osteopenia and importantly the presence of decreased bone mass is associated with an increased risk of fracture [58, 59]. The bone loss has been ascribed to spinal immobility [60]. However, other studies [61, 62] have demonstrated that osteopenia may occur, even in the absence of bony ankylosis, and the authors speculated that the osteopenia was attributable to local effects of inflammatory mediators released from the sites of joint pathology, rather than immobilization. Of interest, Marzo-Ortega et al. [63] demonstrated that BMD improves in patients with SpA who show clinical responses to the TNF-α blocker, etanercept.

## Conclusions

In summary, under physiological conditions, bone remodeling is initiated by activation of osteoclast-mediated bone resorption followed by a phase of osteoblast-mediated bone formation. These cellular processes are tightly “coupled” such that bone structure and mass are preserved during the remodeling cycle. In the SpAs, the cellular machinery of the remodeling unit is co-opted by inflammatory cells and their products, resulting in de-regulation of the remodeling process and uncoupling of bone resorption and formation. A unique feature of the SpAs in comparison to RA is the presence of enhanced bone formation at sites of inflammation. In part, this may be related to the localization of the inflammatory process to the entheses, but, in addition, there is evidence that the cytokine profiles and regulation of the Wnt/βcatenin pathways in the SpAs may differ from the regulatory pathways in RA and other forms of inflammatory arthritis. Many studies have observed that, although inhibition of inflammation with anti-TNF therapy reduces progression of bone erosions at sites of inflammation, enhanced bone formation may persist. The mechanisms involved in this apparent dissociation of inflammation and bone formation have not been fully elucidated. Further studies are needed to confirm these observations and to integrate the findings into improving the treatment protocols for management of the SpAs.
